# General Streptococcus

**Published:** 1918-11

**Authors:** 


### GENERAL STREPTOCOCCUS

Measured by the number of soldiers who are rendered incapable of serving in the line on account of respiratory infections, and the number who have died from this cause, General Streptococcus and his staff, consisting of Major-Generals Pneumococcus and Meningococcus, Brigadier-Generals Influenza and Measles, with their myriad hordes dealing death and destruction, are inflicting greater losses among the American soldiers and are more dangerous enemies than those led by Hindenburg and Ludendorff. This statement is borne out by the call-to-arms to the medical officers of the American Expeditionary Forces in a recent bulletin issued from the Chief Surgeon's Office, which makes the following startling announcement :

" Pneumonia [due most frequently to the streptococcus haemolyticus] is causing more deaths than all other diseases in the American Expeditionary Forces put together. Together with influenza, colds and meningitis it causes more days' loss than all other diseases and injuries combined. Diseases always cause more loss of life and days' service than do battle casualties in every Army. "

Not unlike the Hun, who kills helpless women and children by means of submarines and air-bombs, and who, before making an attack on an army, must render his antagonists defenseless by poisonous gases, General Streptococcus quails before an opponent in his full strength. Therefore the man who is in good physical condition need not fear even the most virulent types of streptococci; but, when this cowardly germ finds a man in a lowered state of vitality through the agency of one of his two vassals, influenza and measles, or from overwork, cold, or dissipation, then he finds the weak spot in the defense of his victims and makes a fierce and, too often, a successful onslaught. Influenza, measles, insufficient air-space and unhygienic living, all make up the artillery which fires the barrage heralding an attack by General Streptococcus Haemolyticus and his death-dealing hordes.

General Streptococcus' favorite victims, next to soldiers in hospitals, are mothers with babes at their breasts, who have trusted themselves during the sacredness of their parturition to careless doctors or ignorant midwives. In contemplating the ravages of General Streptococcus, the writer recalls the lines of Prentice on " The Closing Year " which seem appropriate to this great destroyer :

> " In its swift course it waved its sceptre
> O'er the beautiful, and they were not.
> It laid its pallid hand upon the strong man,
> And the haughty form is fallen
> And the flashing eye is dim.
> It came and faded like a wreath of mist at eve,
> Yet ere it melted on the viewless air,
> It heralded its millions to their homes
> In the dim land of dreams. "

### Military Hospitals Are the Strongholds of General Streptococcus.

General Streptococcus' battle shots cannot penetrate the skin, nor can they destroy healthy tissues. It is only when the natural defenses of the body are broken down that he manages to do his deadly work. Surgeons in military hospitals know that General Streptococcus Haemolyticus is the Kaiser's best friend, for the great majority of those injured in battle by shot and shell would soon recover and return to the line were it not for the deadly interference of General Streptococcus and his infective minions. It may be added, however, that General Streptococcus, although belonging to the Kaiser's class of enemies to mankind, is not under the control of the Potsdam monarch, because during this war he has claimed as victims millions of Germans who have been wounded by the Allied troops; and from reports of the high death rates from influenza and pneumonia among the German troops and the civil population of Germany during the present pandemic, General Streptococcus is playing havoc with the Huns at this time.

The German espionage system is only excelled by that of the Kaiser's most effective ally, General Streptococcus, whose agents are everywhere. Crowded, badly ventilated quarters are places where the enemies that cause respiratory infections, including General Streptococcus Haemolyticus, appear in strongest array. There is much evidence to prove that soldiers in hospitals, whose powers of resistance are lowered by bronchitis, influenza, and measles, or by battle wounds, are secondarily attacked by the great destroyer, General Streptococcus, more than when housed in single open tents or in well-ventilated dwellings.

General Streptococcus Haemolyticus, the arch-field in broncho-pneumonia and empyema, is the same that gains entrance into wounds and causes septicemia, and he seems strongly entrenched in military hospitals. Last year, in some of the cantonment hospitals in the United States, surgeons discontinued operating upon clean cases like hernias, during the epidemics of pneumonia, because it was impossible to perform any operation without interference by this insidious villain. Therefore the strongest fight against General Streptococcus must be made in hospitals. Medical officers, nurses, and male attendants should see to it that every soldier with a cough is isolated, and that every particle of sputum from such patients is destroyed, just as the conscientious surgeon will see to it that a patient with a streptococcus infection, or with erysipelas, is completely isolated, and that everything which comes in contact with an infected wound is destroyed or sterilized.

### The Medical Department leads the Fight against General Streptococcus.

In this fight against General Streptococcus, and his countless hordes, the Medical Department is more important than the artillery or any other branch of military service, though every soldier in the army should be trained to fight germs as well as Germans. Medical officers must first learn the strength and tactics of the enemies comprising the armies of acute respiratory infections; and the plans of campaign against them must be as carefully prepared as those followed by General Foch and his armies in the military strategy that is daily driving the Boche out of France and Belgium. They should see to it taht each soldier in France is drilled over and over again in the performance of his part in the fight to the death with the enemies which he cannot see, but which are more dangerous than the Germans under Hindenburg and Ludendorff.

Each soldier should be taught that he must be singlehanded in the struggle, in addition to fighting these enemies in companies and regimental formations. The American soldier has proved himself more than a match for even the best of German fighters, but if he allows himself to be surrounded by a company or a platoon of enemy troops, he must surrender or be killed. General Streptococcus, like the German soldier, puts up a sorry fight unless he is backed by numbers, but when he has an overwhelming force behind him, he is relentless in his destruction.

Knowing that the armies of General Streptococcus, Major-Generals Pneumococcus and Meningococcus, Brigadier-Generals Influenza and Measles, are our greatest enemies, the American Expeditionary Forces must organize a campaign against them, that must not be relaxed until these invaders have evacuated all the territory which they occupy; and this means that every military hospital, except the isolation or infectious wards, must be freed from this enemy. Even after German militarism has been crushed and peace has been declared, the fight must be kept up against General Streptococcus, because he is no respecter of treaties. He is like " Time, the Tomb Builder ":

> " Remorseless Time, fierce spirit of the Scythe,
> What power can stay him in his silent course,
> Or melt his iron heart with pity?
> Dark, stern, all pitiless, he pauses not,
> Amidst the mighty wrecks that strew his path,
> To sit and muse, like other conquerors,
> Upon the fearful ruin he has wrought. "

